# Toward Reliable Dipole Moments without Single Excitations: The Role of Orbital Rotations and Dynamical Correlation

**DOI:** 10.1021/acs.jctc.4c00471

**Published:** 2024-05-29

**Authors:** Rahul Chakraborty, Matheus Morato F. de Moraes, Katharina Boguslawski, Artur Nowak, Julian Świerczyński, Paweł Tecmer

**Affiliations:** † Institute of Physics, Faculty of Physics, Astronomy, and Informatics, Nicolaus Copernicus University in Toruń, Grudziadzka 5, 87-100 Toruń, Poland; ‡ Institute of Engineering and Technology, Faculty of Physics, Astronomy, and Informatics, Nicolaus Copernicus University in Toruń, Grudzia̧dzka 5, 87-100 Toruń, Poland

## Abstract

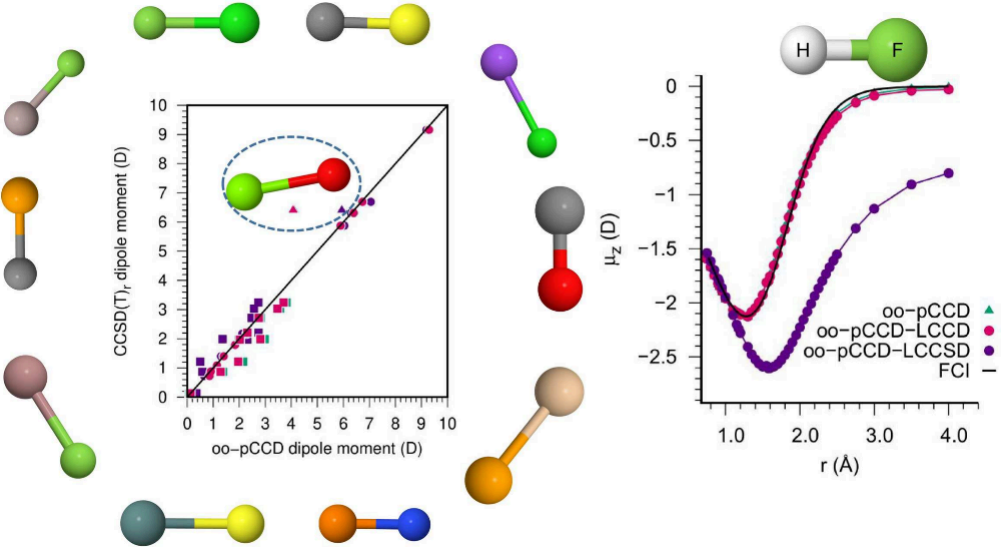

The dipole moment is a crucial molecular property linked to a molecular system’s bond polarity and overall electronic structure. To that end, the electronic dipole moment, which results from the electron density of a system, is often used to assess the accuracy and reliability of new electronic structure methods. This work analyses electronic dipole moments computed with the pair coupled cluster doubles (pCCD) ansätze and its linearized coupled cluster (pCCD-LCC) corrections using the canonical Hartree–Fock and pCCD-optimized (localized) orbital bases. The accuracy of pCCD-based dipole moments is assessed against experimental and CCSD(T) reference values using relaxed and unrelaxed density matrices and different basis set sizes. Our test set comprises molecules of various bonding patterns and electronic structures, exposing pCCD-based methods to a wide range of electron correlation effects. Additionally, we investigate the performance of pCCD-in-DFT dipole moments of some model complexes. Finally, our work indicates the importance of orbital relaxation in the pCCD model and shows the limitations of the linearized couple cluster corrections in predicting electronic dipole moments of multiple-bonded systems. Most importantly, pCCD with a linearized CCD correction can reproduce the dipole moment surfaces in singly bonded molecules, which are comparable to the multireference ones.

## 1 Introduction

The electric dipole moment is the major component of electrostatic interactions, which plays a significant role in many areas of chemistry, physics, and biology.^1^ The electronic component of the molecular dipole moment contains many finer details about the electronic structure and bonding patterns in molecules^2^ and contributes to interpreting spectroscopic data.^3,4^ Dipole moment surfaces, on the other hand, provide information about the change in bond polarity,^5^ intensities of the rovibrational transitions^6^. The reliable determination of this fundamental property is, thus, of preliminary importance for both experimental and theoretical domains. To that end, the quantum chemical modeling of electronic dipole moment provides a common testing ground for approximate wave function models.^7−11^ They can be compared with experimental results that are readily available for many small molecules. For example, the dipole moment was benchmarked against quantum chemical methods like Hartree–Fock theory, second-order Møller–Plesset (MP2) perturbation theory, coupled-cluster (CC) methods, multireference methods, and density functional theory (DFT) approximations.^12,13^ Specifically, coupled cluster-based ansätze have been extensively tested for dipole moment properties^14,15^ and remain an active research field.^16,17^ Maroulis and co-workers^18−24^ performed numerous coupled cluster based studies, including the quantum chemistry gold standard—coupled cluster singles and doubles with perturbative triples (CCSD(T)), on electronic properties of different system types ranging from small di- and triatomic to organic molecules.^25^ Studies by Mazziotti and co-workers^26−28^ have shown alternate routes for evaluation of electric properties using variational reduced density matrices. The elimination of the need for any reference wave function in this approach has great promise for determining electric property in systems with multireference characters. Ground and excited state dipole moments of full configuration interaction (FCI) quality can be reproduced with configuration interaction using a perturbative selection made iteratively (CIPSI) algorithm.^29−31^

Although the electric dipole moment can be easily determined through density matrices, its sensitivity toward the accuracy of the electron density poses a real challenge to various quantum chemical methods.^32−34^ First, orbital relaxation has been shown to have a profound role in this regard.^15,35^ Second, some molecules require the inclusion of triple (or higher) excitations in the wave function expansion to obtain reliable dipole moments.^36,37^ The above aspects are the source of the well-known struggle approximate quantum chemistry methods face in an accurate description of the dipole moment of the CO molecule.^5,38−43^

There are new families of geminal-based methods^34,44−49^ that are yet to be thoroughly tested for dipole moment properties. Some of the most promising ones are those based on the pCCD ansätze.^50−53^ They have seen recent successes in treating strongly correlated systems with mean-field-like scaling. pCCD has the feature of using its optimized orbital basis without defining active spaces.^51,54−56^ The size-extensive and size-consistent nature of orbital-optimized pCCD has motivated a wide range of studies for covalent molecules,^54−63^ noncovalent systems,^64,65^ and excited states,^66−69^ including organic systems.^70,71^ Perturbation theory-based, and linearized coupled cluster (LCC) corrections have also been successfully added to the pCCD wave function to improve the description of dynamic correlation.^72−77^

To the best of our knowledge, little is known about the performance of the pCCD family of methods for ground-state electronic properties like dipole moments. However, there have been studies for such properties with the antisymmetric product of strongly orthogonal geminals (APSG)^78,79^ and other pair-coupled approximate wave function methods. The natural orbital functional theory formulated by Piris and co-workers (PNOFi, i = 1,6) is noteworthy in this respect.^80,81^ Specifically, the PNOF5 is similar to the APSG approach^82^ and, thus, indirectly related to pCCD.^53^ The coupled electron pair approximation(0) (CEPA(0)) and its orbital optimized variant^83^ have similarities with the LCC approach. CEPA-based methods have been tested for dipole moments of various molecules.^84−86^

This work aims to assess the performance of pCCD-type methods in quantifying the electric dipole moments of diatomics of the main group elements, and some larger complexes. The selected diatomic systems represent various bonding patterns (metal–nonmetal, nonmetal–nonmetal, metalloid–nonmetal, metal–metal van der Waals interaction). However, the pCCD framework restricts us to molecules with singlet ground states. Our work focuses on the effects of orbital optimization within pCCD and the inclusion of dynamic correlation. We use linearized coupled-cluster methods for the latter on top of the Hartree–Fock and pCCD wave function: doubles (pCCD-LCCD) and singles and doubles (pCCD-LCCSD) models.^87,88^ Furthermore, we probe the sensitivity of pCCD-based methods for dipole moments regarding different basis set sizes. We compare our electronic dipole moment values with the CCSD and CCSD(T) methods using relaxed and unrelaxed density matrices and experimental values. CCSD(T) has been well tested against dipole moments for a range of chemical specie like molecules of main group elements^89^ and for transition metal compounds.^90^ Specifically, in the large-scale benchmarking study by Liu et al., the average error for CCSD(T) dipole moment with respect to experimental values was found to be ≈0.15 D, showing even better performance for molecules with only main-group atoms. Finally, we extend our studies to pCCD-based static embedding calculations, where we obtain the embedding potential through the DFT approach (pCCD-in-DFT).^91,92^ Precisely, we assess the performance of the pCCD-in-DFT embedding model for the electronic dipole moments of weakly hydrogen-bonded binary complexes such as CO– HF, CO– HCl, N_2_– HF, N_2_– HCl, and the H_2_O···Rg [Rg = He, Ne, Ar, Kr] van der Waals complexes. The electronic structures of these complexes have been studied with various quantum chemical methods and thus represent a good reference point.^93−96^ Additionally, the weak interactions present in these molecules provide a good testing ground for the static embedding approach. In summary, this work reports the performance of some unique pCCD-based models (with and without orbital optimization) with and without dynamic energy corrections for dipole moment calculations.

## 2 Theory

### 2.1 pCCD and Related Methods

Limiting the cluster operator to pair-excitations in the coupled cluster ansätze produces the pCCD ansätze,
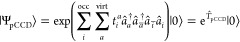
1where  and  ( and ) are the creation and annihilation operators for α-spin (and β-spin) electrons.  is the pair-excitation cluster operator and  is a reference independent particle model, usually the Hartree–Fock wave function. The pCCD model misses a significant fraction of the dynamic electron correlation effects. In this work, we use a posteriori linearized coupled cluster^87^ (LCC) corrections on top of the pCCD wave function to compensate for that. In the LCC correction, the exponential coupled cluster ansätze with a pCCD reference wave function is used as
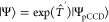
2where  is a cluster operator containing excitation operators  of various levels. The ″′″ in the cluster operator indicates that the pair excitations present in pCCD are excluded. The corresponding energy equation is
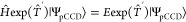
3where  is the electronic Hamiltonian of the system. In the LCC framework, the associated Baker–Campbell–Hausdorff expansion is restricted to the second term, i.e.,
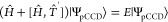
4When we include both single and double excitations (for the pCCD-LCCSD model),  reads,
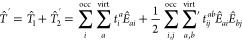
5where
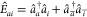
6is the singlet excitation operator. Note that the ″′″ in the second sum of the above equation excludes the cases where *i* = *j* and simultaneously *a* = *b*, while terms where *i* = *j* ∧ *a* ≠ *b* or *i* ≠ *j* ∧ *a* = *b* are still included. Elimination of  amplitudes from  in eq 5 leads to the pCCD-LCCD model. Both pCCD-LCC variants have been successfully used for various molecules, providing a moderate balance between dynamic and nondynamic electron correlation effects.^62,64,75,87,97^

### 2.2 Density Matrices from pCCD and Related Methods

Elements of the 1-electron reduced density matrix (1-RDM) obtained from any wave function Ψ can be expressed as
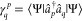
7

For truncated CC models, the 1-electron molecular response properties are calculated using the derivative approach as a response to a small external perturbation related to the property in question (such as dipole moments). In this approach, the response density matrices are often used.^98−101^ Accordingly, elements of the pCCD response 1-RDM are defined as
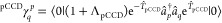
8where  is the electron-pair de-excitation operator.

On the other hand, the response 1-RDM from the pCCD-LCC wave functions can be constructed using the reference response 1-RDM of pCCD from eq 8 and the correlation contribution of the LCC correction on top of the pCCD wave function calculated using the so-called Λ-equations,^88^
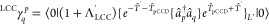
9where  or , for the LCCSD and LCCD models respectively, and
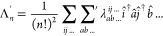
10is the de-excitation operator, where all electron-pair de-excitation are to be excluded as they do not enter the LCC equations (again, indicated by the ″′″). For the LCC response density matrices, only terms that are at most linear in  and  are to be considered. This is indicated by {...}_*L*′_ in Eq. 9. The final 1-RDM from oo-pCCD-LCC(S)D approaches is the sum of the relaxed oo-pCCD and unrelaxed LCC(S)D contributions,
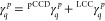
11As evident from eq 8,  contains both the contribution from the reference determinant and the pCCD-correlation part, , while  accounts for the LCC correlation part only. It is to be noted that orbital relaxation due to the LCC correction is not considered in this work.

### 2.3 Dipole Moment Calculation

The total dipole moment of a molecule is defined as
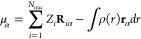
12where the first term accounts for nuclear and the second for electronic contributions. In eq 12, α denotes the axial direction (x, y or z), *Z*_*i*_ charge of the i-th nucleus, *N*_nuc_ the number of nuclei in the molecular structure, and **R** and **r** correspond to the nuclear and electronic coordinates, respectively.

After introducing an atomic orbital (AO) basis set, one α-component of the dipole moment is evaluated from
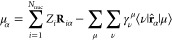
13where  is the density matrix in the AO and  are the dipole moment integrals expressed in the AO basis {χ_ν_}.^102^ Since all pCCD-based methods work in the molecular orbital (MO) basis and hence the corresponding 1-RDMs are defined for the molecular orbitals, we need to perform an AO-MO transformation step of the dipole moment integrals or the 1-RDMs, respectively.

## 3 Computational Details

### 3.1 Structures

The geometries of the main group diatomic molecules were taken from Liu et al.^103^ and references therein. Their bond lengths are collected in Table S1 of the SI. Each diatomic molecule is placed along the *z*-axis.

**Figure 1:**
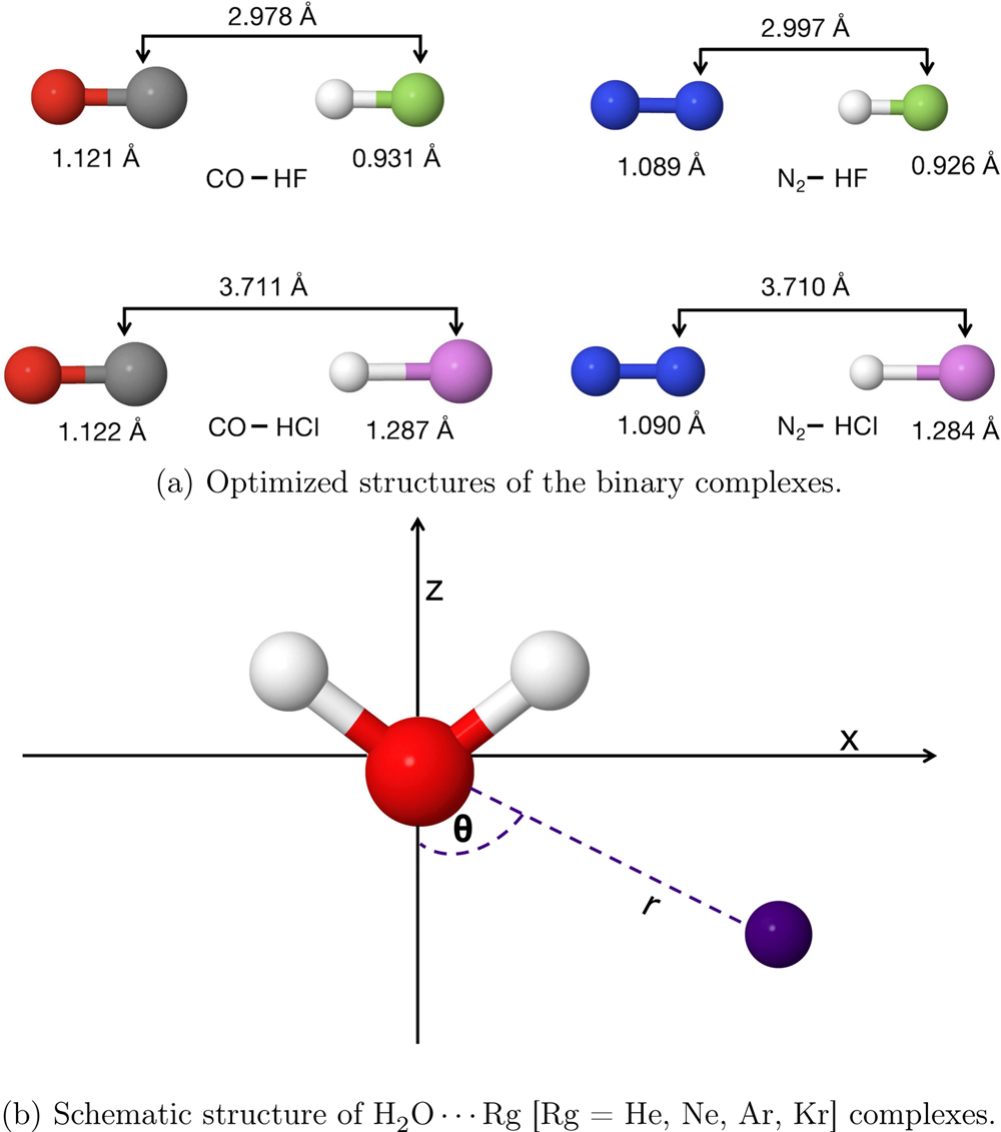
Structural representations of the complexes studied in this work.

The structures of the CO– HF, CO– HCl, N_2_– HF, and N_2_– HCl^93,104,105^ were optimized with the CCSD(T) method and the augmented Dunning-type correlation consistent basis sets of quadruple-ζ quality (aug-cc-pVQZ).^106,107^ The molecules were placed along the *z*-axis, as shown in Figure 1a along with the optimized bond lengths.

The bond parameters of the H_2_O···Rg [Rg = He, Ne, Ar, Kr] complexes were taken from Haskopoulos et al.^96^ Following the original work, these complexes were kept in the xz plane with the center of mass of H_2_O at the origin and the oxygen atom on the negative *z*-axis (see Figure 1b). The equilibrium bond parameters of these 4 complexes are given in Table S8 of the SI.

### 3.2 pCCD-Based Dipole Moment

The pCCD-based dipole moment calculations were carried out in a developer version (v1.4.0dev) of the PYBEST software package.^61,108,109^ The dipole moments were calculated with the Dunning family of basis sets with and without augmentation, that is, (aug-)cc-pVnZ, for n = D, T and Q with optimized general contractions.^106,107,110,111^ Henceforth, the orbital optimized pCCD and the LCC corrections on top of it are called oo-pCCD and oo-pCCD-LCC(S)D. Consequently, the pCCD and pCCD-LCC(S)D will refer to pCCD and a posteriori LCC corrections within a canonical (Hartree–Fock (HF)) orbital basis.

Cholesky decomposition of the two-electron repulsion integrals with a threshold of 10^–5^ was used for all systems. Pipek–Mezey orbital localization^112^ was used to speed up the orbital optimization process for all systems. In all pCCD and oo-pCCD based calculations, all nonvalence orbitals were kept frozen to match the MOLPRO reference results (vide infra).

#### 3.2.1 pCCD-in-DFT

The embedding potentials were generated within the Amsterdam Modeling Suite (AMS2022)^113−115^ and then extracted with the help of the PyADF^116^ scripting framework. In all DFT-in-DFT calculations, the triple-ζ double polarization (TZ2P) basis set,^117^ the PW91^118,119^ exchange–correlation functional, and the PW91k^120^ kinetic energy functional were used. More details about the DFT-in-DFT frozen density embedding (FDE) setup used here to obtain the embedding potential are described in our previous work.^92^ For each embedding calculation, two sets of calculations were performed, in which the system and environment were swapped, and their dipole moment results were added together.

### 3.3 Reference Dipole Moment Calculations

All reference values were obtained using the MOLPRO package version 19.^121−123^ The reference dipole moments were obtained using the CCSD and CCSD(T) methods^124−127^ (relaxed and unrelaxed density matrices) and the same family of basis sets used in pCCD and oo-pCCD based calculations with PyBEST. In this work, CCSD_*u*_ and CCSD(T)_*u*_ refer to dipole moments with unrelaxed densities, whereas CCSD_*r*_ and CCSD(T)_*r*_ are for the same with relaxed densities. The CCSD and CCSD(T) dipole moments are calculated with the CC response formalism as implemented in the MOLPRO software package.

## 4 Results and Discussion

### 4.1 Dipole Moment of Main Group Diatomics

#### 4.1.1 Statistical Analysis

We start our analysis with the diatomic molecules and the basis set dependence. Table S2 of the SI collects all the dipole moments computed with different quantum chemistry methods and (aug-)cc-pVnZ [n = D, T, Q] basis sets with and without augmented functions. All basis sets provide qualitatively similar results. The most significant differences are observed between the cc-pVDZ and cc-pVTZ basis sets, and between the standard and augmented series. The differences within the augmented series are significantly smaller. Table S3 collects the mean unsigned errors (MUE) and root-mean-square errors (RMSE) for all the methods considered in this work in all basis sets with respect to experimental dipole moments. We observe that triple-ζ and quadruple-ζ basis sets produce similar errors. MUE and RMSE increase slightly from aug-cc-pVTZ to aug-cc-pVQZ for oo-pCCD and oo-pCCD-LCCD. However, the opposite is seen for oo-pCCD-LCCSD. In short, not much accuracy is gained by increasing the size of the basis set from triple-ζ to quadruple-ζ in terms of dipole moments, as has been observed in previous works with traditional coupled cluster methods.^12^ To that end, we used the aug-cc-pVTZ as the basis set of choice for further investigations. In addition, we should stress that the dipole moment results are more or less independent of the frozen core approximation (cf. Table S4 of the SI).

Table 1 summarizes the MUE and the RMSE of our pCCD-based methods with respect to the experimental data and the reference theoretical CCSD(T)_*r*_ and CCSD(T)_*u*_ values. The data from Table 1 shows that, on average, the orbital optimization within the pCCD reference function improves the overall performance of the pCCD-based dipole moments with respect to experiment and reference theoretical data. Including LCC on top of pCCD further refines the dipole moment values toward the reference. From a numerical perspective, the MUEs for pCCD and pCCD-LCCSD improve by ≈0.1 D upon the addition of orbital optimization. However, pCCD-LCCD statistics do not show much improvement with the same.

**Table 1:** Error Analysis for the Dataset of 20 Main Group Diatomics Studied in This Work^a^

					CCSD(T)_*r*_							
	Exp.		CCSD(T)_*u*_		full data set		singly bonded		multiply bonded		w/o MgO	
Method	MUE	RMSE	MUE	RMSE	MUE	RMSE	MUE	RMSE	MUE	RMSE	MUE	RMSE
pCCD	0.437	0.640	0.471	0.782	0.393	0.585	0.131	0.155	0.577	0.807	0.437	0.512
pCCD-LCCD	0.356	0.535	0.365	0.663	0.288	0.460	0.091	0.109	0.429	0.638	0.309	0.374
pCCD-LCCSD	0.530	0.753	0.382	0.547	0.465	0.730	0.105	0.138	0.754	1.024	0.585	0.672
CCSD_*u*_	0.180	0.276	0.136	0.307	0.063	0.102	0.032	0.061	0.086	0.131	0.059	0.068
CCSD(T)_*u*_	0.194	0.300	—	—	0.087	0.217	0.011	0.016	0.149	0.306	0.070	0.081
oo-pCCD	0.363	0.604	0.323	0.525	0.336	0.637	0.078	0.097	0.550	0.896	0.375	0.526
oo-pCCD-LCCD	0.345	0.581	0.284	0.474	0.302	0.605	0.068	0.081	0.493	0.852	0.309	0.429
oo-pCCD-LCCSD	0.373	0.476	0.248	0.307	0.283	0.352	0.092	0.136	0.399	0.465	0.393	0.441
CCSD_*r*_	0.191	0.262	0.169	0.289	0.085	0.119	0.038	0.062	0.122	0.156	0.116	0.144

a Errors are calculated in Debye using the aug-cc-pVTZ basis for all methods. MUE and RMSE stand for mean unsigned error  and root mean square error , respectively, where *N* is the number of molecules in the dataset. For CCSD(T)_*r*_ reference data, MUE and RMSE are divided for the full data set, all singly-bonded, and multiply-bonded systems (with and without the outlier MgO).

**Figure 2:**
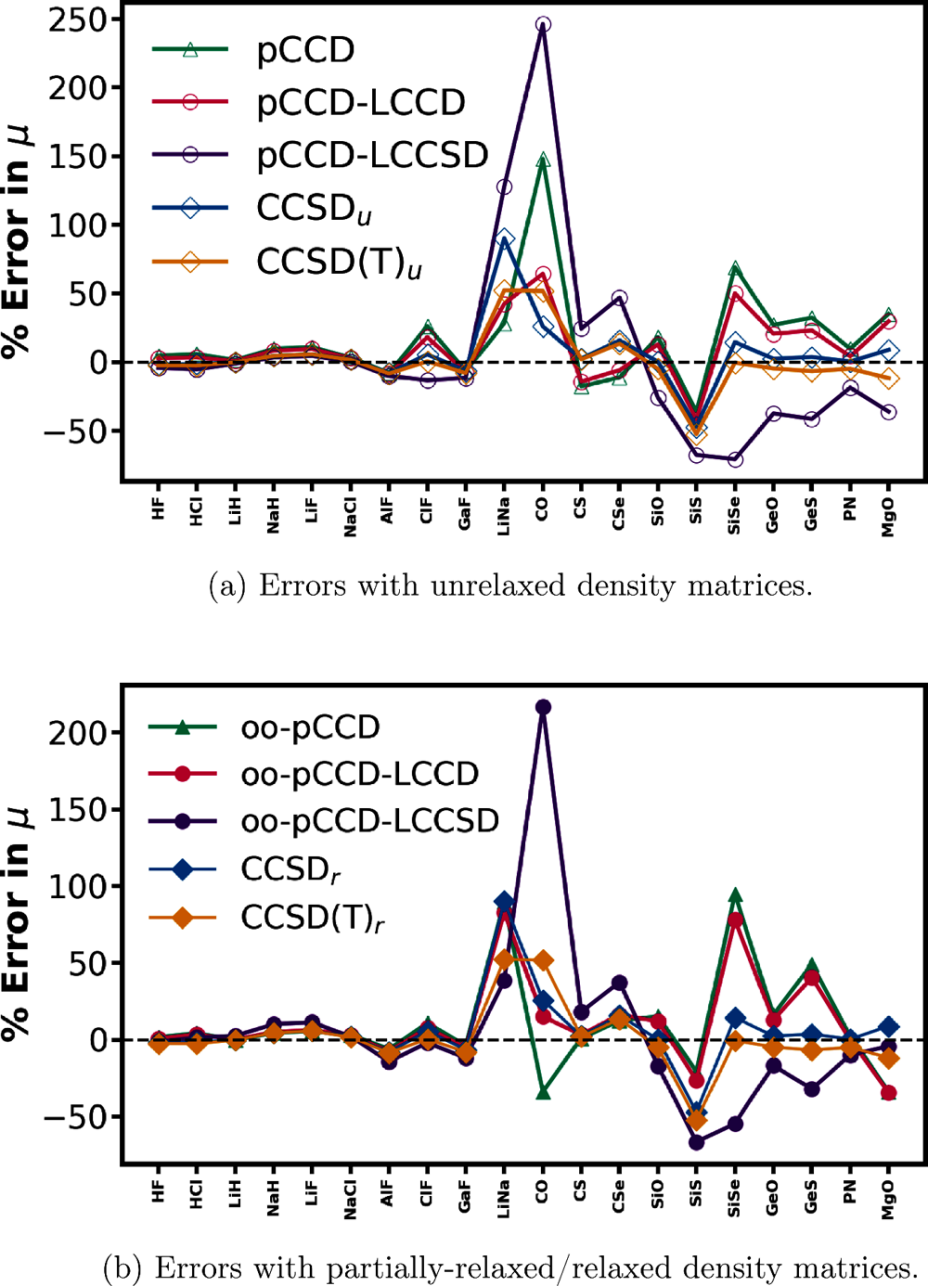
Percentage errors in all methods using aug-cc-pVTZ basis with respect to the experimental dipole moment values for all molecules in the data set.

**Figure 3:**
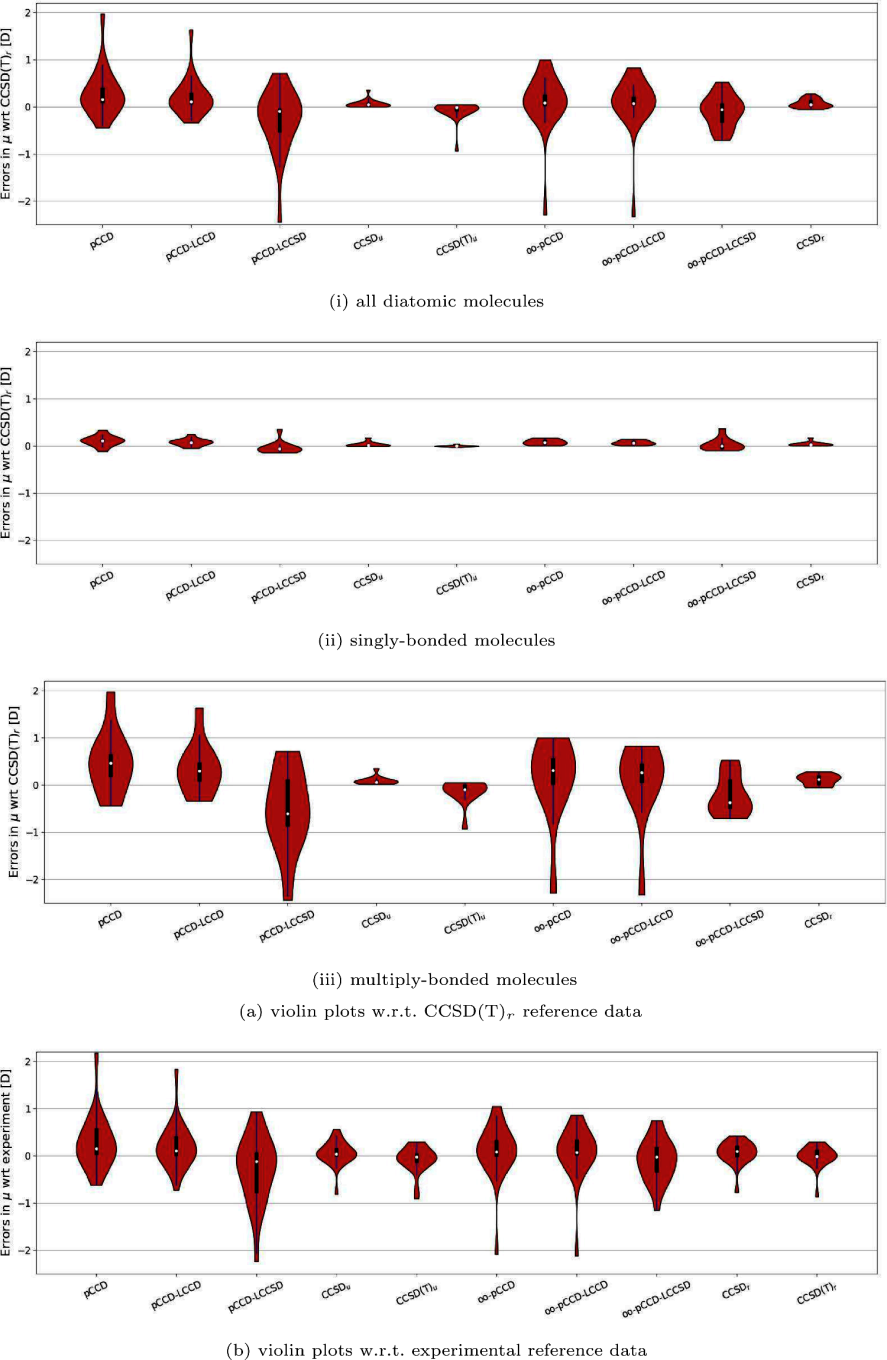
Violin plots illustrating errors (in D) derived from selected methods (refer to Table S2 for numerical values). All errors are reported relative to either (a) CCSD(T)_*r*_ or (b) experimental reference data. A dot in each violin plot represents the median value, while the blue line indicates the 1.5 interquartile range and the black bar the quartile range, respectively.

In Figure 2, we show the percentage errors (with sign) in dipole moments obtained with pCCD-based methods for individual molecules, with respect to the experimental values. Figure 2a shows the performance of pCCD and its variants without orbital optimization, i.e., with completely unrelaxed densities, whereas Figure 2b depicts the same for oo-pCCD and subsequent LCC variants, with relaxed densities achieved through orbital optimization within pCCD. Here, it is important to remember that the oo-pCCD-LCC density matrices are only partially relaxed. In this plot, we see a clear distinction between the behavior of simple singly bonded molecules and the molecules with significant multiple-bond characters. As evident from Figure 2b, the second class of molecules shows higher relative errors with all pCCD-based methods. We also observe that LCCD values remain in close vicinities of the pCCD ones for most of the molecules. Exceptions to this occur for molecules, again, with multiple bond characters (see also last columns in Table 1). The LCCSD values, on the other hand, differ significantly from their counterparts for almost all molecules. Of particular interest is the MgO molecule, where the oo-pCCD-LCCSD seems to perform even better than CCSD(T)_*r*_ with respect to the experiment. The impact of the character of the bond on the dipole moment values obtained with pCCD-based methods is also evident in the violin plots in Figure 3. Specifically, Figure 3a and Figure 3b show the distribution (skewness) of the errors in dipole moments with pCCD-based methods with respect to CCSD(T)_*r*_ and experimental values, respectively. As can be seen, the multiply bonded molecules show a significantly higher spread of errors than the singly bonded molecules. For the latter, the interquartile ranges are distributed closely around the median. If the orbitals are optimized within pCCD, the median and spread are shifted closer to the reference. Moreover, an LCCSD correction introduces outliers and features a broader interquartile range. For multiply bonded systems, the skewness of errors is right-shifted for (oo-)pCCD and (oo)-pCCD-LCCD, while (oo-)-pCCD-LCCSD yields left-shifted ones. Furthermore, (oo)-pCCD-LCCD reduces the interquartile range and shifts the median closer to the reference, while (oo)-pCCD-LCCSD introduces a strong asymmetry, moving the median below the reference point.

Overall, though our statistical analysis shows the utility of adding dynamic correlation with LCC corrections in the pCCD framework, a case-by-case analysis reveals that this is not a black-box tool for all molecules regarding the calculation of dipole moments. That motivates us to conduct a deeper analysis of the performance of pCCD-based methods for different types of molecules and bonding patterns in the next section.

#### 4.1.2 In-Depth Comparison with Reference Theoretical Methods

**Figure 4:**
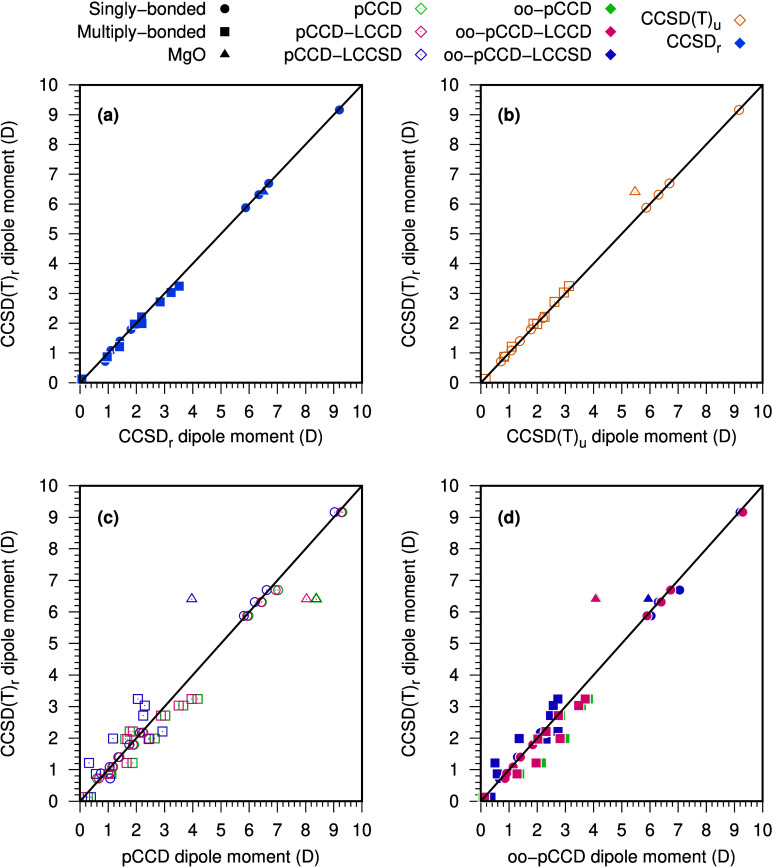
Correlation between the reference CCSD(T)_*r*_ dipole moments (in D) and other CC-based methods. (a) relaxed CCSD; (b) unrelaxed CCSD(T); (c) pCCD and pCCD with LCC corrections; and (d) oo-pCCD and oo-pCCD with LCC corrections.

Figure 4a shows the correlation between the reference CCSD(T)_*r*_ and the CCSD_*r*_ dipole moments (both with relaxed density matrices). We observe an excellent agreement between the two methods for singly bonded molecules (represented by circles). The correlation worsens for multiply bonded systems (marked by squares), underlining the importance of triple excitations. Figure 4b shows good agreement between CCSD(T) results using relaxed and unrelaxed density matrices. The only exception is the MgO molecule (denoted by a triangular shape in Figure 4), for which relaxation has a more profound effect.

By comparing the pCCD-based dipole moments with CCSD(T)_*r*_, we observe a set of characteristic features for each molecule type. Molecules with negligible relaxation effects and triple excitations dependence (mainly singly bonded) provide a very satisfactory agreement between all pCCD-based methods and reference results (cf. Figure 4c-d). Although the variation among pCCD-based methods is slight, we note that the pCCD-LCCSD variant using the canonical orbitals leads to the smallest errors. On the contrary, when orbital-optimized pCCD orbitals are employed, the LCCD correction is the most reliable and results in the smallest errors. Surprisingly, the LCCSD correction on top of oo-pCCD increases the error in some cases.

The MgO molecule presents the most challenging test case for pCCD-LCCD and pCCD-LCCSD methods (cf. Figure 4c). The oo-pCCD-LCCD dipole moment is similar to the pCCD-LCCSD using canonical HF orbitals, which suggests that the orbital relaxation has recovered the effect of the linearized single excitations (compare Figures 4c and 4d). With the LCCSD correction on top of the oo-pCCD, the dipole moment agreement with the CCSD(T)_r_ reference value improves significantly. Specifically, the absolute (and relative) error in the MgO dipole moment reduces from 2.23 D (36%) to 0.46 D (7%) when moving from oo-pCCD-LCCD to oo-pCCD-LCCSD, respectively.

The diatomic molecules with a large contribution of triple excitations to the dipole moment show a similar, but smaller, swing in dipole values between the pCCD-LCCD and pCCD-LCCSD as the one seen for the MgO molecule. However, as the main change in dipole moments is not due to an orbital relaxation effect, the oo-pCCD variation leads to a dipole value closer to the pCCD than the pCCD-LCCSD one. Consequently, the oo-pCCD-LCCD and oo-pCCD-LCCSD results approach the reference from opposite directions. Although the orbital optimization improves the results, the oo-pCCD-LCCD and oo-pCCD-LCCSD dipole moment values have similar but substantial errors. The only exception is the carbon-containing compounds; in these cases, the oo-pCCD-LCCSD error to the CCSD(T)_*r*_ is higher than the oo-pCCD-LCCD. Once some of the studied systems require triple excitations, as concluded during the analysis of Figure 4a, none of the investigated pCCD-based approaches can recover this effect, and, therefore, such an error is expected.

Based on this analysis, the variation of dipole moment values among pCCD, oo-pCCD, and pCCD-LCCSD results can be used to estimate the magnitude of the orbital relaxation and triple excitations for the dipole moment. Systems, where the three values agree with each other have a small dependence on orbital relaxation and triple excitations. Thus, either the pCCD-LCCSD or oo-pCCD-LCCD leads to minor errors with respect to the CCSD(T)_*r*_ reference, that is, a relative average error of around 4%. When oo-pCCD and pCCD-LCCSD are similar, orbital relaxation is required, and the oo-pCCD-LCCSD value should be preferable. Lastly, for distinct oo-pCCD and pCCD-LCCSD values, pCCD-based methods would require a larger excitation order to be reliable. In these cases, excluding the carbon-containing molecules, both oo-pCCD-LCCD and oo-pCCD-LCCSD methods have a relative average error of around 30%. Including the carbon-based ones, the oo-pCCD-LCCD error decreases to 21%, while the oo-pCCD-LCCSD one increases up to 43%.

### 4.2 Dipole Moment Surfaces with pCCD-Based Methods

Dipole moment surfaces (DMS) are essential for estimating rovibrational spectroscopic parameters of molecules. Here, we focus on the DMS of two main group diatomic molecules, HF and CO. Their DMSs have been widely studied^5,128^ in previous theoretical works and, thus, represent suitable test cases for the investigated pCCD-based methods in different bond length regions. In this work, the diatomics AB are placed along the *z*-axis with A (the less electronegative atom) at the origin and B on the positive *z*-axis. Then, the bond between the two atoms of AB is stretched along the positive *z*-axis for constructing the DMS. Hence, a positive μ_*z*_ value will indicate A^–^B^+^ polarity, whereas a negative μ_*z*_ indicates the same as A^+^B^–^.

#### 4.2.1 Hydrogen Fluoride (HF)

**Figure 5:**
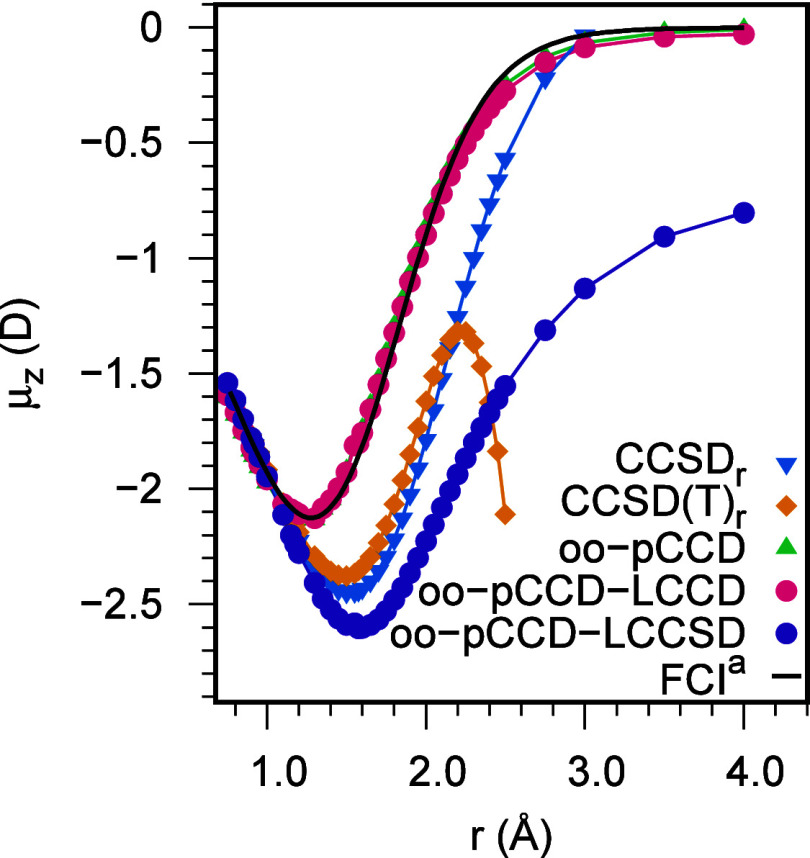
Dipole moment surface of HF in aug-cc-pVTZ basis. ^*a*^FCI/cc-pVDZ DMS is taken from Samanta and Köhn.^129^

Figure 5 shows the DMS of the HF molecule in the aug-cc-pVTZ basis, calculated with oo-pCCD-based methods. We also included the CCSD and CCSD(T) DMSs (both with relaxed densities, i.e., CCSD_*r*_ and CCSD(T)_*r*_) and the FCI DMS (determined for the cc-pVDZ basis set)^129^ for comparison. Around the equilibrium distance (*r*_e_ = 0.917 Å), all oo-pCCD variants agree well with CCSD_*r*_ and CCSD(T)_*r*_, as discussed for singly bonded systems in section 4.1. Passed that region, significant deviations are observed between the curves of oo-pCCD variants and the conventional CC curves. Orbital relaxation has become essential in that region. The CCSD_*r*_ and CCSD(T)_*r*_ dipole moment values significantly deviate from the FCI results. As discussed by Samanta and Köhn,^129^ in this region, the CCSD is unable to compensate the ionic contribution of the Hartree–Fock reference wave function. Although the inclusion of full triple excitations (CCSDT) can improve the CCSD poor modeling, it is not a reasonable zeroth-order wave function for the inclusion of triple excitations perturbatively. This poor description by CC methods during bond-stretching is reinforced by the change in the DMS behavior beyond 2.00 Å and the lack of convergence of coupled perturbed Hartree–Fock (CPHF) calculations for CCSD(T)_*r*_ at 2.25 Å. Therefore, the CCSD(T)_*r*_ dipole moment values are not reliable beyond this point.

The oo-pCCD and oo-pCCD-LCCD DMS lie on top of each other for almost the entire bond length region, indicating the lower significance of the doubles correction on top of pCCD. In good agreement with the previous FCI results,^129^ both the oo-pCCD and oo-pCCD-LCCD dipole moment curves show turnings at around 1.30–1.35 Å and present a much shallower DMS compared to the other methods from Figure 5. These results indicate that the oo-pCCD and oo-pCCD-LCCD can model the HF dipole moment at the bond stretching and dissociation regions. Both have the right shape at larger interatomic distances and converge to the proper asymptotic limit. The oo-pCCD-LCCSD curve, on the other hand, overlaps with the CC curves to a slightly longer bond distance. It also turns at a greater bond length (around 1.60–1.65 Å), showing closer agreement with the turning of CC curves (around 1.50–1.55 Å). At stretched bond lengths, the oo-pCCD-LCCSD curve remains below the CC curve and does not converge to the correct asymptotic limit. That indicates that the linearized singles correction on top of the oo-pCCD wave function modifies the dipole toward the CCSD results but overshoots it at stretched geometries.

#### 4.2.2 Carbon Monoxide (CO)

**Figure 6:**
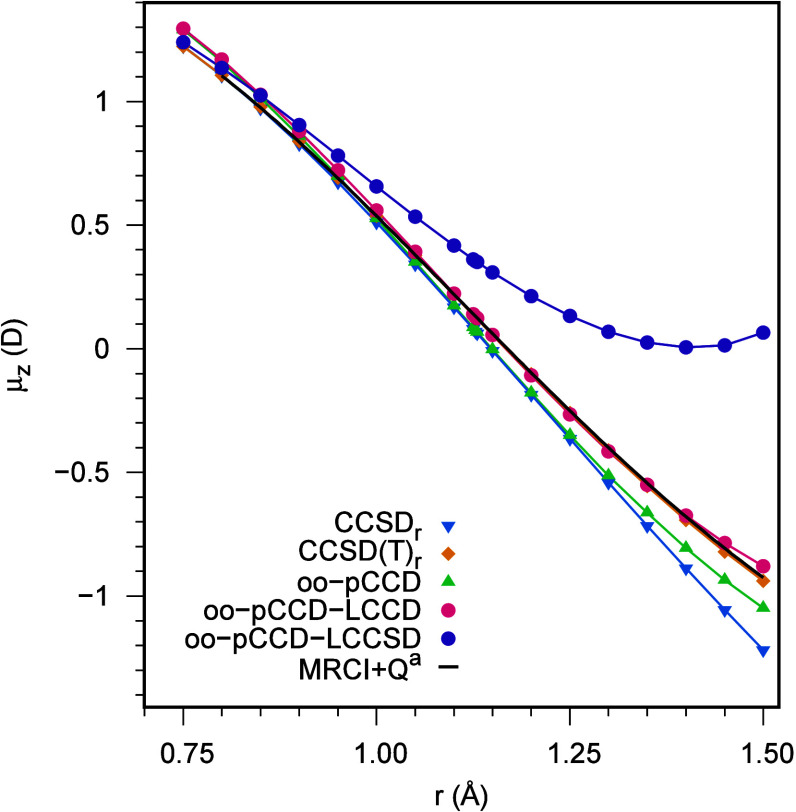
Dipole moment surface of CO in aug-cc-pVTZ basis. ^*a*^The MRCI+Q/aug-cc-pCV6Z values have been taken from Balashov et al.^130^

We focused on the region from 0.75 to 1.50 Å in the CO DMS study. The HF and pCCD wave function optimization beyond that region is very challenging^62^ and will likely not provide reliable dipole moments. In this range of interest of internuclear distances, the CCSD(T)_*r*_ shows a remarkable agreement with the fitted MRCISD+Q dipole values using the finite-field approach and aug-cc-pCV6D basis set^130^ as shown in Figure 6. As discussed in section 4.1, for the CO case, triple excitations are relevant from the equilibrium distance (around 1.13 Å) onward. That is indicated by the growing splitting between CCSD_*r*_ and CCSD(T)_*r*_ dipole values in Figure 6.

Similar to what we observed for HF curves, oo-pCCD-LCCSD overestimates the CO dipole value for large equilibrium distances and has small errors only at the repulsive region (see Figure 5). To that end, the oo-pCCD-LCCSD DMS of CO is not reliable. On the other hand, the oo-pCCD DMS matches the CCSD_*r*_ from 0.90 to 1.25 Å and the oo-pCCD-LCCD DMS resembles the shape of CCSD(T)_*r*_ up to 1.28 Å. Throughout the internuclear distances, the average absolute error in the dipole moment of oo-pCCD-LCCD compared to the MRCI+Q reference is about 0.023 D (or around 4% considering relative errors). Thus, the oo-pCCD-LCCD provides comparable DMS with the computationally more expensive multireference and CCSD(T)_*r*_ calculations.

### 4.3 Dipole Moments from pCCD-Based Static Embedding

Dipole moments are often used to assess the performance of DFT-based embedding approaches.^131^ The calculated dipole moments are susceptible to electron density changes caused by environmental effects and, thus, are valuable measures for validating the quality of the embedding potential.^132,133^ To that end, we investigate the performance of recently implemented pCCD-in-DFT static embedding models^92^ for two sets of weakly interacting systems: linear hydrogen-bonded binary complexes and coplanar water complexes with noble gases. Their structural parameters are presented in Figures 1a and 1b, respectively. Building on the experience gained in the previous section and knowing the importance of orbital relaxation in oo-pCCD, we solely focused on orbital-optimized variants. The supramolecular oo-pCCD-LCCSD dipole moments show low error with respect to the CCSD(T)_*r*_ data (shown in Table S9 of the SI) and, thus, provide a reliable supramolecular reference except for CO-HF and CO-HCl, where oo-pCCD-LCCD performs better, similarly to the observer for the isolated CO molecule in section 4.2.2.

**Figure 7:**
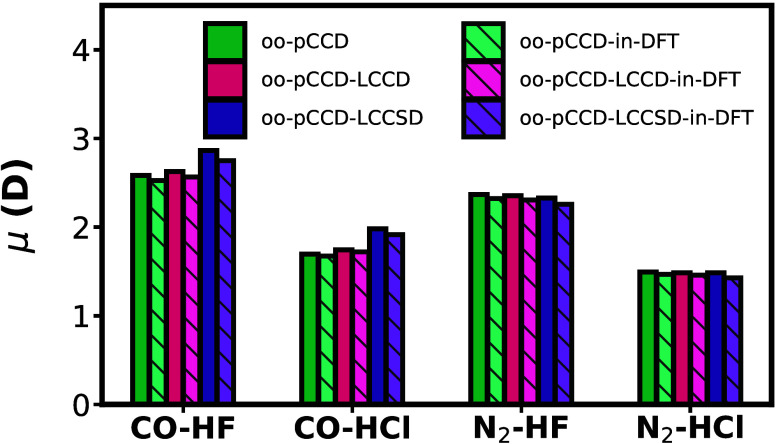
Dipole moments (μ in D) of the binary complexes from oo-pCCD variants and the corresponding embedding approaches in the aug-cc-pVTZ basis set.

Table 2 collects dipole moments obtained from various pCCD models with and without embedding and the difference between them. Figure 7 summarizes the performance of the orbital optimized pCCD-based embedding models for dipole moments of weakly hydrogen-bonded complexes (the binary complexes, see also Figure 1a). The static embedding approach produces dipole moments closer to the respective supramolecular values with both oo-pCCD and oo-pCCD-LCC methods. Interestingly, the difference in embedding and supramolecular dipole moment values is lower with oo-pCCD and oo-pCCD-LCCD compared to oo-pCCD-LCCSD. This is most likely attributed to the limitations of oo-pCCD-LCCSD when individual fragments possess multiple bonds, as we observed for the diatomics (vide supra).

**Table 2:** Dipole Moment (μ in D) from aug-cc-pVTZ oo-pCCD and oo-pCCD-in-DFT Types of Methods and Their Differences^a^

	oo-pCCD			oo-pCCD-LCCD			oo-pCCD-LCCSD		
Complex	supra.	emb.	error	supra.	emb.	error	supra.	emb.	error
CO–HF	2.586	2.528	–0.058	2.630	2.569	–0.061	2.866	2.752	–0.114
CO–HCl	1.698	1.677	–0.021	1.745	1.723	–0.022	1.983	1.918	–0.065
N_2_–HF	2.371	2.325	–0.046	2.357	2.309	–0.048	2.329	2.260	–0.069
N_2_–HCl	1.497	1.471	–0.026	1.488	1.461	–0.027	1.488	1.431	–0.057
H_2_O···He	1.928	1.928	0.000	1.910	1.910	0.000	1.836	1.836	0.000
H_2_O···Ne	1.918	1.918	0.000	1.899	1.900	0.001	1.821	1.824	0.003
H_2_O···Ar	1.905	1.904	–0.001	1.887	1.885	–0.002	1.810	1.810	0.000
H_2_O···Kr	1.940	1.948	0.008	1.922	1.930	0.008	1.863	1.856	–0.007

a The errors are calculated as μ_emb._ – μ_supra._.

**Figure 8:**
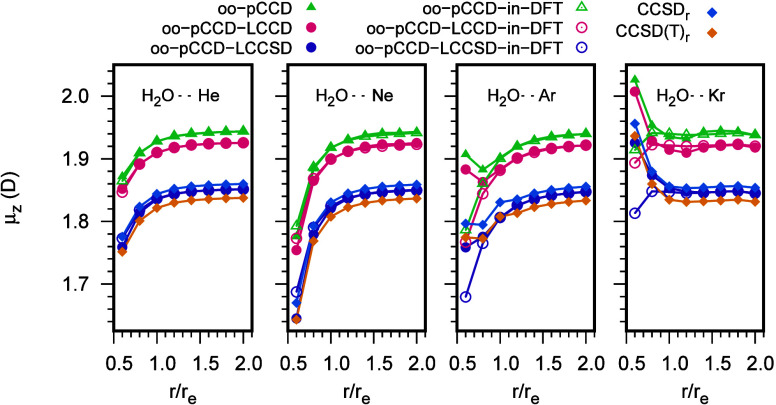
Distance dependence of the calculated dipole moment components of the H_2_O···Rg [Rg = He, Ne, Ar, and Kr] complexes in aug-cc-pVTZ basis.

We also study the dipole moments of the van der Waal’s complexes between H_2_O and the first four inert gases. Here, the performance of the static embedding approach is even better for all oo-pCCD variants. This is to be expected as, for these complexes, the electronic properties are dominated by the highly polar H_2_O molecule, and it is easier to estimate them with embedding. As far as the supramolecular results in comparison to CCSD(T)_*r*_ are concerned, oo-pCCD-LCCSD shows the best performance, with errors comparable to CCSD_*r*_ (bottom part of Table S9 of the SI). Most importantly, the changes in the dipole moment with change in the inert gas molecule (decrease from He to Ar and then increase for Kr) are captured by all oo-pCCD-based methods (supramolecular and embedding). Figure 8 shows the change in dipole moments of the H_2_O···Rg complexes with the distance between H_2_O and the inert gas atom. For these curves, the distance between H_2_O and the Rg atom is increased in multiples of the equilibrium distances, keeping the angles the same for the respective structures. Here, we plot the major component of the dipole, that is μ_*z*_. A plot for μ_*x*_ is shown in Figure S2 of the SI. For H_2_O···He and H_2_O···Ne, the supramolecular trends in the changes in the dipole are well-reproduced by the embedding methods throughout the distances scanned. For H_2_O···Ar and H_2_O···Kr, the embedding methods differ from the supramolecular variants significantly at shorter distances. We anticipate that this is caused by the shortcoming of the kinetic energy functional, which has been observed for other complexes with Ar and Kr.^132^ The nonparallelity errors (difference between highest error and lowest error between embedding and supramolecular curves) are 0.121, 0.116, and 0.079 (H_2_O···Ar), and 0.112, 0.114, and 0.112 (H_2_O···Kr) for oo-pCCD-in-DFT, oo-pCCD-LCCD-in-DFT, and oo-pCCD-LCCSD-in-DFT respectively.

Barring the initial points for H_2_O···Ar and H_2_O···Kr, the oo-pCCD-LCCSD curves (both supra and embedding) are between those of CCSD_*r*_ and CCSD(T)_*r*_ for all systems. To conclude, the performance of both oo-pCCD-LCCSD and oo-pCCD-LCCSD-in-DFT is encouraging for these systems, keeping in mind the low computational cost of the static embedding approach.

## 5 Conclusions and Outlook

In this work, we investigated the performance of various pCCD-based methods for predicting dipole moments. Our study shows that orbital optimization is essential and improves the overall performance of pCCD-based methods. Altogether, the best performance is obtained for the oo-pCCD-LCCD method, which is comparable to CCSD in predicting dipole moments. Specifically, oo-pCCD-LCCD approaches CCSD accuracy in dipole moments for singly bonded systems, while it reproduces the DMSs obtained by multireference methods. Thus, we demonstrated that reliable dipole moments can also be obtained without explicitly including single excitations in the wave function model.

For equilibrium structures, oo-pCCD-LCCD provides good agreement with the CCSD(T)_*r*_ dipole moment values for singly bonded systems—for instance, HF, AlF, and LiNa. For multiply bonded systems (such as SiO, GeS, and PN), the oo-pCCD-LCCD performance deteriorates (errors w.r.t. CCSD(T)_*r*_ are up to around 30%). The only exception is systems containing the carbon atom, where the relative errors drop below 5%. The oo-pCCD-LCCD approach is also noticeably good in the modeling of DMSs. Specifically, for the HF molecule, oo-pCCD-LCCD provides excellent agreement with FCI even in the region where CCSD (and CCSD(T)) fail. For carbon monoxide (up to a distance of 1.50 Å), the agreement among oo-pCCD-LCCD, CCSD(T)_*r*_, and MRCISD+Q results is remarkable.

On the contrary, the presence of linearized singles in the LCC correction on top of the pCCD reference worsens the performance when multiply bonded diatomic molecules are considered. That is particularly true for the investigated DMSs, where the LCCSD correction provides erroneous dipole moments. The presence of singles, however, improves the description of van der Waals complexes as singles are crucial for dispersion interactions.^64^ All pCCD-in-DFT models provide similar results for supramolecular and embedded dipole moments. As expected, for van der Waals complexes, the oo-pCCD-LCCSD provides the best agreement with coupled cluster reference data.

Finally, this work provides a reference point for further improvements of pCCD-based models. Specifically, our in-depth analysis of dipole moments demonstrates that when oo-pCCD provides a good reference function (like van der Waals and single-bonded systems), the LCCD (for singly bonded systems) and LCCSD (for van der Waals interactions) corrections can improve the electric properties of the system. We point out cases (e.g., multiple-bonded systems) where oo-pCCD does not produce reliable dipole moments, despite giving qualitatively correct potential energy surfaces as observed in previous works.^62,87,134^ For such molecules, LCCD does not improve the overall description, and pCCD-LCCSD tends to overcorrect dipole moments. It remains to be checked if using other than response density matrices (which are linear in nature) will bring some improvements. Furthermore, it needs to be determined whether frozen-pair or tailored variants of pCCD-based models^62^ will correct for deficiencies in the investigated LCC corrections.

## Data Availability

The data underlying this study are available in the published article and its Supporting Information The PyBEST code is available on Zenodo at https://zenodo.org/records/10069179 and on PyPI at https://pypi.org/project/pybest/.

## Supporting Information Available

Experimental dipole moments and bond lengths, dipole moments from different basis sets, dipole moments without a frozen core approximation, data for DMSs of HF and CO, CO potential energy surface, structural parameters for the H_2_O···Rg systems, and additional comparisons and graphs for embedding results (PDF)
