# Supplementary material for: Toward Reliable Dipole Moments without Single Excitations: The Role of Orbital Rotations and Dynamical Correlation
Source: J Chem Theory Comput. 2024 May 29;20(11):4689–702. doi: 10.1021/acs.jctc.4c00471 (PMC11171297; doi:10.1021/acs.jctc.4c00471)
Supplement: Supplementary file 1 — ct4c00471_si_001.pdf [file ct4c00471_si_001.pdf]

Toward Reliable Dipole Moments without Single Excitations: The Role of Orbital Rotations and Dynamical Correlation

Rahul Chakraborty[1], Matheus Morato F. de Moraes[1], Katharina Boguslawski[1], Artur Nowak[1], Julian Świerczyński[2], and Paweł Tecmer[1]

[1]*Institute of Physics, Faculty of Physics, Astronomy and Informatics, Nicolaus Copernicus University in Torun, Grudziadzka 5, 87-100 Torun, Poland*
[2]*Institute of Engineering and Technology, Faculty of Physics, Astronomy, and Informatics, Nicolaus Copernicus University in Toruń, 87-100 Toruń, Poland*

Corresponding authors: matheusmorat@gmail.com, k.boguslawski@fizyka.umk.pl, ptecmer@fizyka.umk.pl

Supplementary Information

Table S1: Experimental bond lengths and dipole moments of diatomics studied in this work.

Type	Molecule	Bond length (Å)	μ (D)
Singly-bonded	HF	0.917	1.827
	HCl	1.275	1.109
	LiH	1.596	5.882
	NaH	1.889	6.400
	LiF	1.564	6.327
	NaCl	2.361	9.002
	AlF	1.654	1.515
	ClF	1.628	0.850
	GaF	1.774	2.400
	LiNa	2.810	0.470
Multiply-bonded	CO	1.128	0.112
	CS	1.535	1.958
	CSe	1.676	1.990
	SiO	1.510	3.098
	SiS	1.730	1.740
	SiSe	2.058	1.100
	GeO	1.625	3.282
	GeS	2.012	2.000
	PN	1.491	2.751
$A^{2+}B^{2-}$	MgO	1.749	6.200

Table S2: Dipole moments (μ in D) of main-group diatomics in different methods. Frozen core approximation has been used here for all the methods. CC_u and CC_r denote unrelaxed and relaxed dipole moments of the corresponding CC methods.

Molecule	Basis	pCCD	pCCD -LCCD	pCCD -LCCSD	$CCSD_u$	$CCSD(T)_u$	oo-pCCD	oo-pCCD -LCCD	oo-pCCD -LCCSD	$CCSD_r$	$CCSD(T)_r$
HF	cc-pVDZ	1.915	1.899	1.814	1.822	1.817	1.861	1.857	1.861	1.830	1.819
	cc-pVTZ	1.915	1.889	1.790	1.813	1.800	1.872	1.861	1.838	1.823	1.807
	cc-pVQZ	1.907	1.880	1.789	1.815	1.801	1.866	1.852	1.832	1.826	1.809
	aug-cc-pVDZ	1.923	1.890	1.746	1.787	1.781	1.857	1.845	1.800	1.803	1.788
	aug-cc-pVTZ	1.915	1.880	1.749	1.794	1.782	1.860	1.845	1.806	1.808	1.791
	aug-cc-pVQZ	1.913	1.878	1.758	1.802	1.788	1.857	1.842	1.815	1.814	1.797
HCl	cc-pVDZ	1.386	1.378	1.339	1.329	1.312	1.344	1.346	1.404	1.323	1.312
	cc-pVTZ	1.242	1.228	1.173	1.184	1.172	1.215	1.211	1.239	1.182	1.174
	cc-pVQZ	1.210	1.195	1.145	1.150	1.139	1.194	1.189	1.196	1.150	1.142
	aug-cc-pVDZ	1.212	1.199	1.126	1.144	1.128	1.198	1.196	1.190	1.146	1.132
	aug-cc-pVTZ	1.175	1.147	1.049	1.093	1.081	1.158	1.149	1.126	1.097	1.086
	aug-cc-pVQZ	1.163	1.138	1.061	1.108	1.096	1.153	1.146	1.139	1.110	1.099
LiH	cc-pVDZ	5.903	5.857	5.681	5.735	5.735	5.721	5.753	5.951	5.735	5.735
	cc-pVTZ	5.931	5.889	5.764	5.848	5.848	5.829	5.855	6.015	5.848	5.848
	cc-pVQZ	5.964	5.924	5.799	5.855	5.855	5.854	5.876	6.025	5.856	5.856

Continued on next page

Table S2 – *Continued from previous page*

Molecule	Basis	pCCD	pCCD -LCCD	pCCD -LCCSD	$CCSD_u$	$CCSD(T)_u$	oo-pCCD	oo-pCCD -LCCD	oo-pCCD -LCCSD	$CCSD_r$	$CCSD(T)_r$
LiH	aug-cc-pVDZ	5.995	5.950	5.782	5.911	5.911	5.912	5.936	6.088	5.912	5.912
	aug-cc-pVTZ	5.972	5.932	5.811	5.872	5.872	5.873	5.894	6.037	5.873	5.873
	aug-cc-pVQZ	5.982	5.944	5.818	5.868	5.868	5.869	5.889	6.031	5.869	5.869
NaH	cc-pVDZ	6.959	6.871	6.557	6.423	6.423	6.610	6.657	7.011	6.424	6.424
	cc-pVTZ	6.959	6.871	6.557	6.615	6.615	6.610	6.657	7.011	6.616	6.616
	cc-pVQZ	7.002	6.913	6.571	6.665	6.665	6.679	6.721	7.049	6.666	6.666
	aug-cc-pVDZ	7.029	6.940	6.533	6.678	6.678	6.679	6.724	7.065	6.679	6.679
	aug-cc-pVTZ	7.028	6.940	6.617	6.692	6.692	6.693	6.734	7.058	6.693	6.693
	aug-cc-pVQZ	7.027	6.940	6.588	6.687	6.687	6.696	6.736	7.052	6.689	6.689
LiF	cc-pVDZ	6.464	6.427	5.991	6.195	6.173	6.363	6.350	6.140	6.232	6.179
	cc-pVTZ	6.406	6.372	6.063	6.273	6.245	6.358	6.345	6.194	6.296	6.251
	cc-pVQZ	6.413	6.384	6.139	6.285	6.260	6.379	6.367	6.247	6.304	6.266
	aug-cc-pVDZ	6.490	6.469	6.250	6.369	6.359	6.461	6.452	6.337	6.389	6.364
	aug-cc-pVTZ	6.451	6.424	6.202	6.322	6.304	6.411	6.399	6.295	6.341	6.311
	aug-cc-pVQZ	6.442	6.415	6.204	6.324	6.304	6.407	6.396	6.317	6.342	6.310
NaCl	cc-pVDZ	9.324	9.264	8.841	9.012	8.949	9.252	9.230	9.038	9.030	8.955
	cc-pVTZ	9.182	9.128	8.834	9.047	8.997	9.201	9.184	9.057	9.057	9.000
	cc-pVQZ	9.225	9.177	8.956	9.138	9.099	9.201	9.194	9.209	9.146	9.100
	aug-cc-pVDZ	9.350	9.321	9.088	9.231	9.199	9.346	9.339	9.225	9.242	9.203
	aug-cc-pVTZ	9.305	9.263	9.028	9.191	9.158	9.305	9.291	9.199	9.200	9.161
	aug-cc-pVQZ	9.264	9.224	9.038	9.179	9.146	9.278	9.266	9.203	9.186	9.148
AlF	cc-pVDZ	1.559	1.498	1.123	1.280	1.261	1.519	1.469	1.111	1.329	1.278
	cc-pVTZ	1.372	1.348	1.226	1.286	1.265	1.375	1.347	1.179	1.314	1.279
	cc-pVQZ	1.383	1.359	1.300	1.366	1.351	1.403	1.379	1.275	1.387	1.365
	aug-cc-pVDZ	1.433	1.418	1.352	1.430	1.431	1.496	1.475	1.314	1.458	1.442
	aug-cc-pVTZ	1.394	1.383	1.359	1.394	1.387	1.424	1.401	1.300	1.416	1.401
	aug-cc-pVQZ	1.370	1.342	1.315	1.400	1.392	1.409	1.387	1.325	1.419	1.406
ClF	cc-pVDZ	1.303	1.265	0.962	1.094	1.073	1.109	1.091	1.059	1.128	1.091
	cc-pVTZ	1.119	1.069	0.799	0.968	0.921	0.979	0.954	0.907	1.002	0.951
	cc-pVQZ	1.059	1.002	0.741	0.915	0.861	0.929	0.900	0.841	0.948	0.895
	aug-cc-pVDZ	1.073	1.013	0.738	0.884	0.865	0.875	0.853	0.832	0.923	0.890
	aug-cc-pVTZ	1.072	1.004	0.738	0.898	0.854	0.943	0.917	0.833	0.934	0.886
	aug-cc-pVQZ	1.072	1.000	0.730	0.896	0.845	0.900	0.872	0.823	0.929	0.879
GaF	cc-pVDZ	2.255	2.192	1.830	2.021	1.963	2.260	2.207	1.844	2.030	1.943
	cc-pVTZ	2.172	2.130	1.926	2.098	2.043	2.216	2.181	1.963	2.079	2.009
	cc-pVQZ	2.198	2.167	2.057	2.195	2.147	2.26	2.228	2.084	2.170	2.112
	aug-cc-pVDZ	2.301	2.281	2.187	2.318	2.290	2.397	2.370	2.172	2.307	2.261
	aug-cc-pVTZ	2.244	2.222	2.122	2.244	2.207	2.309	2.278	2.114	2.219	2.171
	aug-cc-pVQZ	2.224	2.192	2.113	2.251	2.212	2.295	2.265	2.138	2.223	2.176
LiNa	cc-pVDZ	0.515	0.559	0.939	0.898	0.880	0.898	0.869	0.648	0.900	0.879
	cc-pVTZ	0.562	0.600	0.976	0.892	0.721	0.889	0.858	0.651	0.894	0.725
	cc-pVQZ	0.570	0.609	1.001	0.898	0.671	0.895	0.862	0.661	0.900	0.677
	aug-cc-pVDZ	0.558	0.576	0.846	0.892	0.864	0.886	0.857	0.644	0.895	0.863
	aug-cc-pVTZ	0.603	0.670	1.071	0.894	0.716	0.893	0.860	0.652	0.896	0.720
	aug-cc-pVQZ	0.590	0.646	1.069	0.898	0.653	0.901	0.868	0.661	0.900	0.661
CO	cc-pVDZ	0.144	0.069	0.494	0.252	0.262	0.124	0.186	0.474	0.172	0.221
	cc-pVTZ	0.164	0.086	0.446	0.178	0.210	0.089	0.146	0.397	0.106	0.167
	cc-pVQZ	0.189	0.110	0.406	0.135	0.169	0.078	0.132	0.352	0.065	0.125
	aug-cc-pVDZ	0.265	0.165	0.421	0.172	0.182	0.073	0.132	0.386	0.097	0.141

Continued on next page

Table S2 – *Continued from previous page*

Molecule	Basis	pCCD	pCCD -LCCD	pCCD -LCCSD	$CCSD_u$	$CCSD(T)_u$	oo-pCCD	oo-pCCD -LCCD	oo-pCCD -LCCSD	$CCSD_r$	$CCSD(T)_r$
CO	aug-cc-pVTZ	0.278	0.184	0.388	0.141	0.170	0.074	0.129	0.355	0.070	0.126
	aug-cc-pVQZ	0.280	0.191	0.368	0.128	0.161	0.078	0.131	0.344	0.059	0.117
CS	cc-pVDZ	1.583	1.641	2.350	1.935	1.928	1.785	1.860	2.241	1.843	1.879
	cc-pVTZ	1.639	1.692	2.397	1.959	1.949	1.917	1.968	2.273	1.878	1.918
	cc-pVQZ	1.685	1.728	2.419	1.979	1.963	1.963	2.009	2.288	1.898	1.937
	aug-cc-pVDZ	1.615	1.677	2.423	2.021	2.014	1.851	1.924	2.323	1.936	1.971
	aug-cc-pVTZ	1.611	1.678	2.433	2.010	1.999	1.977	2.026	2.313	1.928	1.969
	aug-cc-pVQZ	1.626	1.680	2.416	2.000	1.984	1.983	2.028	2.305	1.919	1.959
CSe	cc-pVDZ	1.838	1.912	2.849	2.255	2.215	2.078	2.176	2.671	2.126	2.148
	cc-pVTZ	1.825	1.908	2.911	2.275	2.233	2.197	2.274	2.726	2.153	2.185
	cc-pVQZ	1.857	1.927	2.927	2.268	2.222	2.226	2.297	2.718	2.146	2.178
	aug-cc-pVDZ	1.793	1.890	2.919	2.335	2.293	2.167	2.262	2.741	2.214	2.234
	aug-cc-pVTZ	1.768	1.871	2.926	2.304	2.260	2.240	2.315	2.737	2.183	2.213
	aug-cc-pVQZ	1.763	1.848	2.878	2.277	2.230	2.238	2.309	2.718	2.155	2.187
SiO	cc-pVDZ	3.309	3.135	1.893	2.534	2.405	3.070	2.944	2.112	2.697	2.506
	cc-pVTZ	3.456	3.297	2.173	2.881	2.698	3.375	3.268	2.390	3.009	2.791
	cc-pVQZ	3.610	3.447	2.310	3.089	2.908	3.531	3.430	2.573	3.207	2.998
	aug-cc-pVDZ	3.613	3.438	2.162	2.976	2.850	3.506	3.398	2.461	3.115	2.933
	aug-cc-pVTZ	3.662	3.502	2.301	3.109	2.943	3.574	3.472	2.572	3.232	3.032
	aug-cc-pVQZ	3.686	3.527	2.343	3.157	2.984	3.584	3.484	2.626	3.273	3.073
SiS	cc-pVDZ	1.016	0.937	0.427	0.731	0.632	1.365	1.238	0.418	0.794	0.684
	cc-pVTZ	1.091	1.002	0.565	0.903	0.801	1.396	1.293	0.564	0.947	0.840
	cc-pVQZ	1.044	0.975	0.646	0.952	0.862	1.380	1.285	0.623	0.993	0.898
	aug-cc-pVDZ	1.088	0.995	0.517	0.853	0.768	1.433	1.325	0.532	0.907	0.815
	aug-cc-pVTZ	1.111	1.010	0.563	0.920	0.829	1.380	1.283	0.582	0.965	0.869
	aug-cc-pVQZ	1.106	1.021	0.638	0.955	0.869	1.292	1.212	0.661	0.997	0.904
SiSe	cc-pVDZ	1.786	1.609	0.362	0.997	0.840	2.174	1.983	0.488	1.167	0.977
	cc-pVTZ	1.786	1.609	0.362	1.243	1.064	2.174	1.983	0.488	1.388	1.183
	cc-pVQZ	1.797	1.628	0.465	1.337	1.166	2.190	2.007	0.571	1.473	1.278
	aug-cc-pVDZ	1.828	1.616	0.278	1.156	1.010	2.254	2.048	0.462	1.310	1.134
	aug-cc-pVTZ	1.861	1.652	0.321	1.261	1.095	2.146	1.960	0.501	1.404	1.212
	aug-cc-pVQZ	1.860	1.659	0.399	1.339	1.171	2.178	1.998	0.573	1.474	1.282
GeO	cc-pVDZ	3.752	3.524	1.697	2.712	2.516	3.214	3.072	2.149	2.926	2.663
	cc-pVTZ	3.893	3.699	2.027	3.092	2.833	3.569	3.451	2.502	3.260	2.959
	cc-pVQZ	4.069	3.857	2.067	3.311	3.051	3.739	3.628	2.697	3.462	3.168
	aug-cc-pVDZ	4.120	3.921	2.175	3.287	3.096	3.805	3.687	2.669	3.467	3.210
	aug-cc-pVTZ	4.168	3.957	2.052	3.364	3.126	3.815	3.703	2.732	3.521	3.241
	aug-cc-pVQZ	4.174	3.961	2.043	3.406	3.157	3.819	3.709	2.776	3.553	3.270
GeS	cc-pVDZ	2.514	2.325	0.964	1.730	1.523	2.900	2.675	1.141	1.896	1.672
	cc-pVTZ	2.625	2.432	1.131	1.993	1.765	2.956	2.779	1.298	2.120	1.887
	cc-pVQZ	2.608	2.436	1.264	2.116	1.902	2.978	2.812	1.419	2.233	2.014
	aug-cc-pVDZ	2.685	2.482	1.133	2.008	1.814	3.070	2.882	1.348	2.151	1.944
	aug-cc-pVTZ	2.646	2.459	1.170	2.075	1.866	2.983	2.812	1.362	2.201	1.985
	aug-cc-pVQZ	2.647	2.470	1.250	2.137	1.929	2.981	2.819	1.435	2.253	2.039
PN	cc-pVDZ	2.578	2.486	1.968	2.394	2.290	2.493	2.422	2.155	2.474	2.368
	cc-pVTZ	2.868	2.736	2.140	2.638	2.482	2.704	2.634	2.377	2.714	2.582
	cc-pVQZ	2.984	2.844	2.247	2.762	2.595	2.795	2.726	2.488	2.833	2.700
	aug-cc-pVDZ	2.931	2.794	2.223	2.688	2.581	2.782	2.715	2.419	2.771	2.660
	aug-cc-pVTZ	3.023	2.871	2.240	2.765	2.614	2.821	2.752	2.478	2.843	2.716

Continued on next page

Table S2 – *Continued from previous page*

Molecule	Basis	pCCD	pCCD -LCCD	pCCD -LCCSD	$CCSD_u$	$CCSD(T)_u$	oo-pCCD	oo-pCCD -LCCD	oo-pCCD -LCCSD	$CCSD_r$	$CCSD(T)_r$
PN	aug-cc-pVQZ	3.062	2.904	2.290	2.807	2.643	2.830	2.762	2.523	2.880	2.749
MgO	cc-pVDZ	8.366	8.041	4.423	4.934	3.838	4.158	4.122	5.758	4.600	4.952
	cc-pVTZ	8.377	8.036	3.962	5.996	4.696	4.109	4.075	5.940	5.721	5.706
	cc-pVQZ	8.430	8.089	3.752	6.576	5.256	3.949	3.923	5.996	6.316	6.196
	aug-cc-pVDZ	8.366	8.041	4.423	6.573	5.341	4.158	4.122	5.758	6.349	6.294
	aug-cc-pVTZ	8.377	8.036	3.962	6.759	5.470	4.109	4.075	5.940	6.513	6.404
	aug-cc-pVQZ	8.430	8.089	3.752	6.911	5.608	3.949	3.923	5.996	6.664	6.510

Table S3: Error analysis w.r.t. experimental values for the dataset of 20 main group diatomics studied in this work. Errors are calculated as $|\mu_{\text{Method}}$ - $\mu_{\text{Exp.}}|$

Quantity	Basis	pCCD	pCCD -LCCD	pCCD -LCCSD	$CCSD_u$	$CCSD(T)_u$	oo-pCCD	oo-pCCD -LCCD	oo-pCCD -LCCSD	$CCSD_r$	$CCSD(T)_r$
MUE	cc-pVDZ	0.385	0.325	0.600	0.303	0.399	0.334	0.347	0.493	0.278	0.313
	cc-pVTZ	0.387	0.316	0.552	0.181	0.273	0.339	0.325	0.409	0.183	0.204
	cc-pVQZ	0.412	0.336	0.520	0.169	0.203	0.363	0.348	0.365	0.181	0.143
	aug-cc-pVDZ	0.426	0.344	0.498	0.170	0.222	0.355	0.337	0.403	0.164	0.157
	aug-cc-pVTZ	0.437	0.356	0.530	0.180	0.194	0.363	0.345	0.373	0.191	0.148
	aug-cc-pVQZ	0.442	0.363	0.520	0.193	0.173	0.376	0.357	0.349	0.205	0.143
RMSE	cc-pVDZ	0.599	0.513	0.787	0.441	0.655	0.574	0.560	0.610	0.457	0.452
	cc-pVTZ	0.601	0.506	0.766	0.260	0.442	0.585	0.565	0.512	0.266	0.290
	cc-pVQZ	0.631	0.531	0.763	0.255	0.322	0.627	0.606	0.463	0.251	0.234
	aug-cc-pVDZ	0.633	0.530	0.688	0.269	0.336	0.607	0.579	0.505	0.252	0.258
	aug-cc-pVTZ	0.640	0.535	0.753	0.276	0.300	0.604	0.581	0.476	0.262	0.244
	aug-cc-pVQZ	0.651	0.545	0.764	0.290	0.271	0.636	0.614	0.447	0.276	0.240

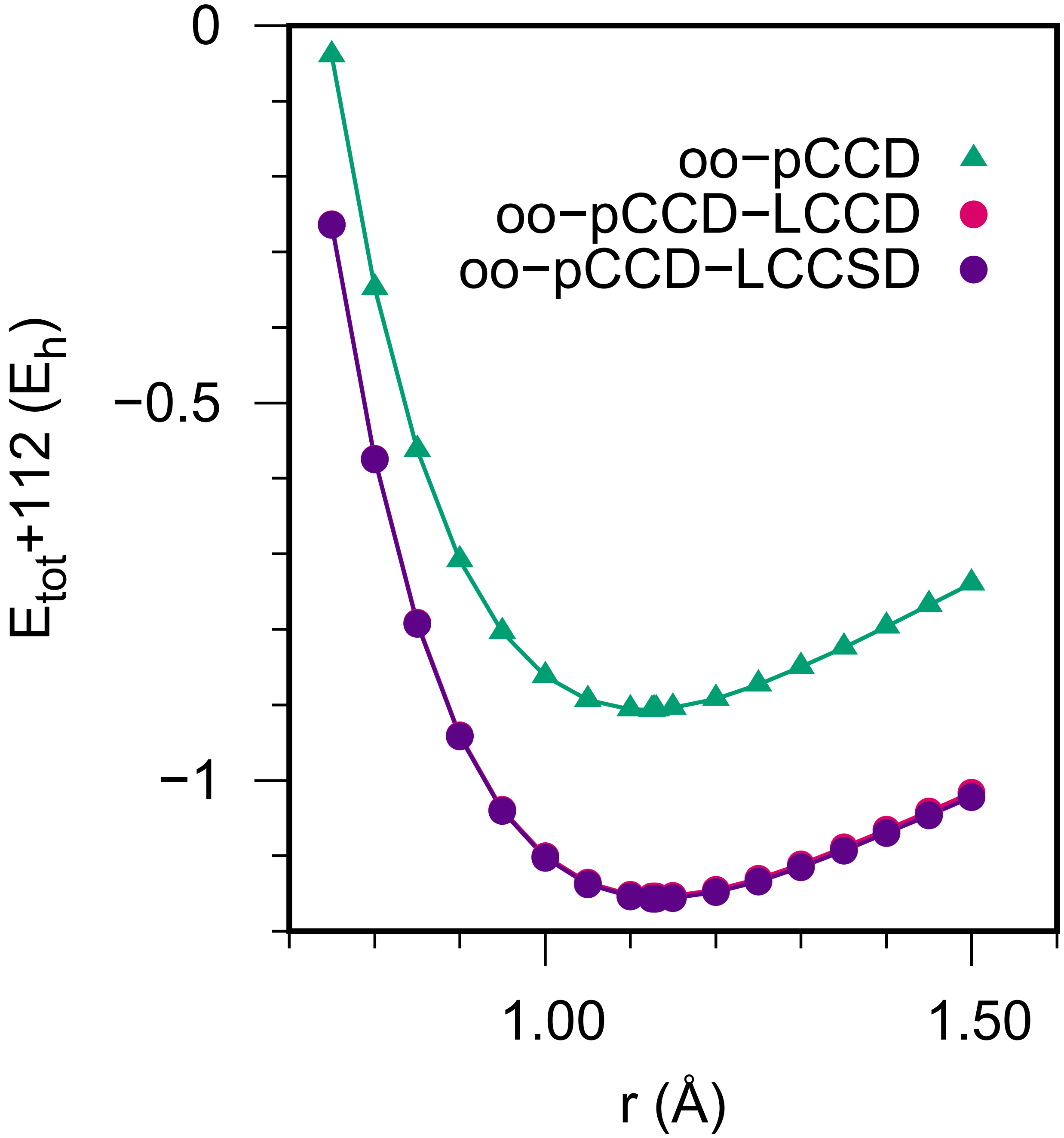

Figure S1: Potential energy surface of CO with oo-pCCD method and its dynamic correlation correction variants in aug-cc-pVTZ basis.

Table S4: Dipole moment (μ in D) of diatomic molecules in aug-cc-pVQZ basis set without frozen core. CC_u and CC_r denote unrelaxed and relaxed dipole moments of the corresponding CC methods.

Species	pCCD	pCCD-LCCD	pCCD-LCCSD	$CCSD_u$	$CCSD(T)_u$	oo-pCCD	oo-pCCD-LCCD	oo-pCCD-LCCSD	$CCSD_r$	$CCSD(T)_r$
HF	1.913	1.878	1.764	1.806	1.792	1.855	1.840	1.819	1.818	1.800
HCl	1.163	1.138	1.055	1.104	1.092	1.151	1.144	1.133	1.107	1.095
LiH	5.984	5.945	5.798	5.854	5.851	5.912	5.927	5.994	5.856	5.851
NaH	7.028	6.946	6.452	6.577	6.551	6.740	6.778	6.960	6.578	6.553
LiF	6.444	6.416	6.186	6.311	6.288	6.413	6.399	6.290	6.328	6.295
NaCl	9.265	9.232	8.990	9.144	9.102	9.285	9.274	9.153	9.154	9.108
AlF	1.371	1.345	1.379	1.439	1.432	1.394	1.373	1.374	1.456	1.446
ClF	1.072	1.001	0.744	0.908	0.853	0.897	0.868	0.839	0.940	0.887
GaF	2.224	2.188	2.090	2.270	2.252	2.295	2.261	2.117	2.309	2.267
LiNa	0.587	0.649	0.800	0.678	0.653	0.897	0.882	0.473	0.676	0.662
CO	0.280	0.191	0.376	0.131	0.166	0.084	0.139	0.352	0.062	0.122
CS	1.629	1.684	2.469	2.024	2.006	1.996	2.043	2.349	1.939	1.981
CSe	1.764	1.850	2.891	2.252	2.204	2.266	2.334	2.710	2.134	2.170
SiO	3.687	3.528	2.378	3.193	3.013	3.686	3.576	2.545	3.304	3.102
SiS	1.107	1.022	0.655	0.976	0.885	1.364	1.273	0.630	1.016	0.922
SiSe	1.899	1.683	0.422	1.428	1.252	2.068	1.892	0.630	1.553	1.350
GeO	4.176	3.957	1.822	3.377	3.114	3.862	3.735	2.401	3.550	3.252
GeS	2.649	2.473	1.056	2.081	1.850	3.081	2.901	1.007	2.214	1.982
PN	3.063	2.907	2.327	2.839	2.664	2.826	2.760	2.559	2.907	2.773
MgO	8.430	8.088	4.139	6.859	5.526	3.510	3.551	5.232	6.660	6.484

Figure S2: Distance dependence of the calculated dipole moment components in aug-cc-pVTZ basis for the $H_2O \cdots Rg$ [Rg = He, Ne, Ar, and Kr] complexes.

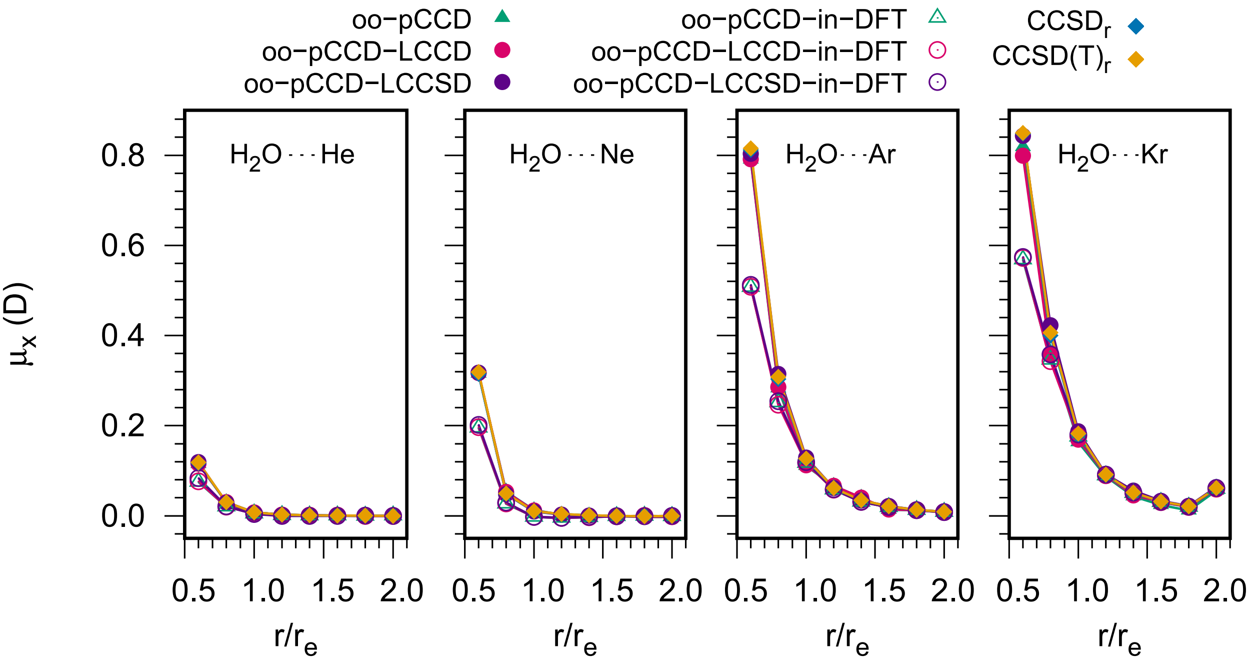

Table S5: Dipole moment surface of the HF molecule in aug-cc-pVTZ basis.

r (Å)	oo-pCCD	oo-pCCD-LCCD	oo-pCCD-LCCSD	$CCSD_r$	$CCSD(T)_r$
0.75	-1.606	-1.594	-1.541	-1.548	-1.538
0.80	-1.681	-1.668	-1.616	-1.625	-1.613
0.85	-1.758	-1.745	-1.697	-1.703	-1.688
0.90	-1.835	-1.820	-1.778	-1.781	-1.765
0.917	-1.860	-1.845	-1.806	-1.808	-1.791
0.95	-1.908	-1.892	-1.862	-1.860	-1.841
1.00	-1.974	-1.958	-1.946	-1.938	-1.917
1.10	-2.080	-2.065	-2.112	-2.088	-2.060
1.15	-2.100	-2.089	-2.200	-2.158	-2.127
1.175	-2.111	-2.100	-2.239	-2.191	-2.158
1.20	-2.118	-2.108	-2.278	-2.223	-2.188
1.30	-2.134	-2.127	-2.407	-2.334	-2.291
1.35	-2.088	-2.085	-2.475	-2.378	-2.330
1.40	-2.048	-2.048	-2.524	-2.412	-2.358
1.45	-1.992	-1.995	-2.563	-2.436	-2.374
1.50	-1.921	-1.928	-2.589	-2.447	-2.377
1.55	-1.801	-1.812	-2.581	-2.446	-2.366
1.575	-1.791	-1.803	-2.605	-2.440	-2.354
1.60	-1.742	-1.756	-2.603	-2.430	-2.338
1.65	-1.637	-1.655	-2.590	-2.399	-2.294
1.70	-1.526	-1.547	-2.565	-2.354	-2.234
1.75	-1.412	-1.436	-2.528	-2.293	-2.158
1.80	-1.296	-1.323	-2.482	-2.217	-2.067
1.85	-1.182	-1.211	-2.426	-2.126	-1.963
1.90	-1.071	-1.102	-2.364	-2.023	-1.851
1.95	-0.965	-0.997	-2.298	-1.909	-1.734
2.00	-0.866	-0.898	-2.227	-1.786	-1.619
2.05	-0.773	-0.806	-2.155	-1.656	-1.512
2.10	-0.687	-0.720	-2.082	-1.523	-1.421
2.15	-0.609	-0.642	-2.009	-1.388	-1.353
2.20	-0.538	-0.571	-1.937	-1.253	-1.317
2.25	-0.474	-0.507	-1.867	-1.122	-1.320
2.30	-0.417	-0.449	-1.799	-0.996	-1.368
2.35	-0.366	-0.397	-1.734	-0.877	-1.469
2.40	-0.321	-0.351	-1.671	-0.765	-1.625
2.45	-0.281	-0.311	-1.611	-0.662	-1.838
2.50	-0.246	-0.275	-1.554	-0.567	-2.110
2.75	-0.127	-0.151	-1.311	-0.219	—
3.00	-0.067	-0.086	-1.131	-0.033	—
3.50	-0.022	-0.040	-0.906	—	—
4.00	-0.009	-0.030	-0.803	—	—

Table S6: Dipole moment surface of the CO molecule in aug-cc-pVTZ basis.

r (Å)	oo-pCCD	oo-pCCD-LCCD	oo-pCCD-LCCSD	$CCSD_r$	$CCSD(T)_r$
0.75	1.291	1.295	1.240	1.224	1.223
0.80	1.162	1.170	1.135	1.105	1.106
0.85	1.014	1.027	1.025	0.972	0.978
0.90	0.860	0.879	0.905	0.828	0.838
0.95	0.697	0.722	0.781	0.674	0.691
1.00	0.527	0.559	0.656	0.511	0.536
1.05	0.352	0.392	0.534	0.342	0.378
1.10	0.174	0.224	0.417	0.169	0.217
1.125	0.085	0.140	0.362	0.081	0.137
1.128	0.074	0.129	0.355	0.070	0.127
1.13	0.068	0.123	0.351	0.063	0.120
1.15	-0.003	0.056	0.309	-0.008	0.056
1.20	-0.179	-0.107	0.213	-0.186	-0.103
1.25	-0.349	-0.265	0.132	-0.364	-0.259
1.30	-0.513	-0.415	0.069	-0.541	-0.410
1.35	-0.664	-0.55	0.025	-0.716	-0.555
1.40	-0.806	-0.675	0.006	-0.887	-0.693
1.45	-0.935	-0.786	0.014	-1.055	-0.821
1.50	-1.048	-0.879	0.066	-1.218	-0.939

Table S7: Potential energy surface of the CO molecule in aug-cc-pVTZ basis.

r (Å)	oo-pCCD	oo-pCCD-LCCD	oo-pCCD-LCCSD
0.75	-112.039847	-112.263221	-112.264186
0.80	-112.347900	-112.573941	-112.574974
0.85	-112.562476	-112.791373	-112.792613
0.90	-112.708294	-112.940263	-112.941638
0.95	-112.803726	-113.038946	-113.040487
1.00	-112.862264	-113.100881	-113.102623
1.05	-112.893868	-113.136013	-113.137993
1.10	-112.905944	-113.151733	-113.153993
1.125	-112.906435	-113.154085	-113.156501
1.128	-112.906275	-113.154165	-113.156602
1.13	-112.906164	-113.15419	-113.156638
1.15	-112.903983	-113.153519	-113.156103
1.20	-112.892097	-113.145475	-113.148426
1.25	-112.873341	-113.130644	-113.134004
1.30	-112.850002	-113.111302	-113.115103
1.35	-112.823797	-113.089152	-113.093311
1.40	-112.796049	-113.065542	-113.070078
1.45	-112.767685	-113.041273	-113.046226
1.50	-112.739429	-113.016702	-113.022521

Table S8: Equilibrium bond parameters of the $H_2O\cdots Rg$ [Rg = He, Ne, Ar, Kr] complexes used in this work. [HM10]

Species	r_e (Å)	θ_e
$H_2O\cdots He$	3.186	79°
$H_2O\cdots Ne$	3.228	76°
$H_2O\cdots Ar$	3.651	100°
$H_2O\cdots Kr$	3.884	109.3°

Table S9: Supramolecular dipole moments (μ in D) of the binary complexes in aug-cc-pVTZ basis. CC_u and CC_r denote unrelaxed and relaxed dipole moments of the corresponding CC methods. The errors with respect to reference $CCSD(T)_r$ values are calculated as $\epsilon = \mu_{\text{Method}} - \mu_{\text{CCSD(T)}_r}$.

Species	oo-pCCD	ϵ	oo-pCCD-LCCD	ϵ	oo-pCCD-LCCSD	ϵ	$CCSD_r$	ϵ	$CCSD(T)_r$
CO−HF	2.586	-0.030	2.630	-0.014	2.866	-0.250	2.571	-0.045	2.616
CO−HCl	1.698	-0.007	1.745	0.040	1.983	0.278	1.652	-0.053	1.705
N_2−HF	2.371	0.047	2.357	0.043	2.329	0.005	2.335	0.011	2.324
N_2−HCl	1.497	0.064	1.488	0.055	1.466	0.033	1.441	0.008	1.433
$H_2O\cdots He$	1.928	0.107	1.910	0.089	1.835	0.032	1.844	0.023	1.821
$H_2O\cdots Ne$	1.918	0.110	1.899	0.091	1.821	0.013	1.831	0.023	1.808
$H_2O\cdots Ar$	1.905	0.104	1.887	0.086	1.810	0.009	1.822	0.021	1.801
$H_2O\cdots Kr$	1.940	0.096	1.922	0.078	1.863	0.019	1.865	0.021	1.844

References

[HM10] Anastasios Haskopoulos and George Maroulis. "Interaction electric hyperpolarizability effects in weakly bound H2O ··· Rg (Rg= He, Ne, Ar, Kr and Xe) complexes." In: *J. Phys. Chem. A* 114.33 (2010), pp. 8730–8741.
